# The impact of bacterial diversity on resistance to biocides in oilfields

**DOI:** 10.1038/s41598-021-02494-7

**Published:** 2021-11-29

**Authors:** Gabriela Feix Pereira, Harry Luiz Pilz-Junior, Gertrudes Corção

**Affiliations:** 1 Department of Microbiology, Immunology and Parasitology, Institute of Basic Health Sciences, Universidade Federal Do Rio Grande Do Sul, Rua Sarmento Leite 500, Porto Alegre, RS 90050-170 Brazil; 2 Dorf Ketal Research and Development Center, Rua da Pedreira 559, Nova Santa Rita, RS 92480-000 Brazil

**Keywords:** Microbiology, Environmental microbiology, Water microbiology

## Abstract

Extreme conditions and the availability of determinate substrates in oil fields promote the growth of a specific microbiome. Sulfate-reducing bacteria (SRB) and acid-producing bacteria (APB) are usually found in these places and can harm important processes due to increases in corrosion rates, biofouling and reservoir biosouring. Biocides such as glutaraldehyde, dibromo-nitrilopropionamide (DBNPA), tetrakis (hydroxymethyl) phosphonium sulfate (THPS) and alkyl dimethyl benzyl ammonium chloride (ADBAC) are commonly used in oil fields to mitigate uncontrolled microbial growth. The aim of this work was to evaluate the differences among microbiome compositions and their resistance to standard biocides in four different Brazilian produced water samples, two from a Southeast Brazil offshore oil field and two from different Northeast Brazil onshore oil fields. Microbiome evaluations were carried out through 16S rRNA amplicon sequencing. To evaluate the biocidal resistance, the Minimum Inhibitory Concentration (MIC) of the standard biocides were analyzed using enriched consortia of SRB and APB from the produced water samples. The data showed important differences in terms of taxonomy but similar functional characterization, indicating the high diversity of the microbiomes. The APB and SRB consortia demonstrated varying resistance levels against the biocides. These results will help to customize biocidal treatments in oil fields.

## Introduction

In oil fields, systems containing water are prone to uncontrolled microbial growth, which may trigger serious problems. Uncontrolled microbial activity can increase oil contamination, microbially induced corrosion, biofouling and the generation of dangerous metabolic byproducts, such as hydrogen sulfide (H_2_S), known as biosouring^1,2^.

In mature fields, the secondary recovery of oil requires water/gas injection to push the oil from the well to the platform surface. To decrease storage costs, produced water generated during oil extraction is commonly used, but it first receives a biocidal treatment to prevent microbial contamination in the well. Among the most employed biocides worldwide used in produced water treatment are glutaraldehyde (27% of the market share), dibromo-nitrilopropionamide (DBNPA; 24% of the market share), tetrakis (hydroxymethyl) phosphonium sulfate (THPS; 9% of the market share) and alkyl dimethyl benzyl ammonium chloride (ADBAC; 3% of the market share)^3^. The Brazilian O&G industry usually employs THPS (batch application) and/or ADBAC (continuous low dosage application) as chemical treatments for produced water^4^.

Due to the extreme environmental conditions found in oil fields, such as high temperature, low oxygen content and high salinity, some specific groups of bacteria are present. Among them, the main biocide targets are acid-producing bacteria (APB), also known as fermentative bacteria, and sulfate-reducing bacteria (SRB)^5^. APB produces several types of organic and inorganic acids and other metabolites under anaerobic conditions. The generated metabolites interact with steel surfaces, affecting electrochemical reactions and, consequently, the corrosion rate. The SRB group, in addition to increasing corrosion, is known to generate H_2_S, a toxic, corrosive and inflammable gas, which causes concerns in terms of oil field structural integrity and worker safety^6^.

The application of similar treatments over the years without technical monitoring selects those microorganisms with high biocide resistance levels^7^. Factors such as microbiome composition, treatment history and resistance level need to be observed to choose the best method for microbial control. Therefore, the aim of this study was to evaluate four different produced water samples in terms of their microbiome compositions and metabolic activities. The Minimum Inhibitory Concentration (MIC) of glutaraldehyde, DBNPA, THPS and ADBAC against SRB and APB consortia obtained from produced water samples were determined.

## Materials and methods

### Produced water samples and biocides

**Figure 1:**
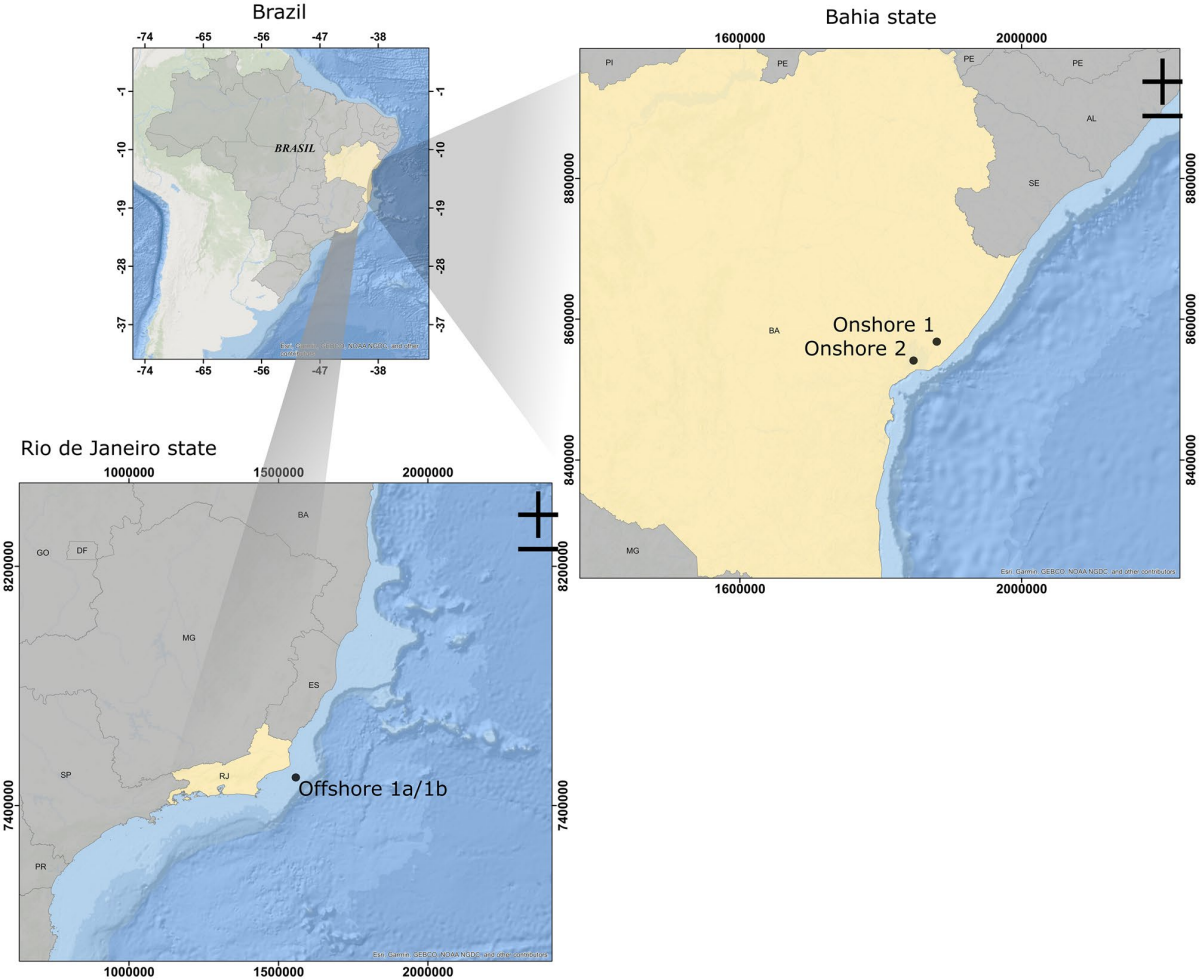
Map of Brazil with detailed locations of the sample collection points in Bahia State (right) and Rio de Janeiro State (botton). Created using the component ArcMap from the software ArcGIS 10.6.1 (https://www.arcgis.com/).

Four samples of produced water from three Brazilian oil fields were used in this study. Two samples were collected in different wells from the same offshore field located in the state of Rio de Janeiro (Offshore 1a and Offshore 1b), and two samples were collected from different fields in the state of Bahia (Onshore 1 and Onshore 2) (Fig. 1). Sampling flasks were filled to the top to avoid oxygen contamination. The samples were kept refrigerated and in the dark during transportation and were filtered immediately after the arrived, within 72 h by a 0.22 µm filter. The filters were maintained at − 20 °C before analysis. Dorf Ketal Brazil Ltda. provided the produced water and biocide samples (glutaraldehyde, DBNPA, THPS and ADBAC).

### Produced water characterization

Produced water samples were characterized immediately after receipt according to salinity, sulfate content, pH and microbial concentration. Salinity (chloride content) was determined by potentiometric titration in a Titrino Plus 848 (Metrohm, Switzerland). The equipment was connected to a silver electrode, and 0.01 mol L^−1^ AgNO_3_ solution was used as the standard. Sulfate quantification in water samples was carried out by the ICP-OES Agilent 5100 technique (Agilent, USA). A sulfur standard was used to determine the sulfate content. pH was determined with a pH meter (Edge, Hanna Instruments). The microbial concentration was determined by ATP quantification using a luminometer (Photonmaster, Luminultra) and specific reagent kit (ATP Quench Gone Aqueous Kit, Luminultra).

### DNA extraction and quality check

Two 100 mL aliquots of each sample were filtered through 0.45 µm membranes to obtain duplicates. Total genomic DNA was extracted from each membrane using a DNEasy PowerSoil Kit (Qiagen, Germany) following the manufacturer’s instructions. The extracted DNA was quantified by a Quantus fluorimeter (Promega, USA).

### 16S rRNA gene amplification and sequencing

The V4 region of the 16S rRNA gene was amplified using primers 515F (5´ GTGCCAGCMGCCGCGGTAA 3´) and R806 (5´ GGACTACHVGGGTWTCTAAT 3’), both modified to contain an Illumina adapter region ^8^. PCR amplification was carried out by mixing ~ 100 ng of genomic DNA, 1.0 mM MgCl_2_, 0.5 µM of each primer, 0.2 mM of dNTP, 2 U Platinum *Taq DNA* Polymerase High Fidelity (Life Technologies, USA), and 1 × reaction buffer. The thermal cycling conditions for amplification were an initial denaturation (2 min at 94 °C) followed by 25 cycles of amplification (45 s at 94 °C, 45 s at 55 °C and 1 min at 72 °C) and a final extension cycle (6 min at 72 °C) in a Mastercycler Personal 5332 Thermocycler (Eppendorf, Germany).

Amplicons were purified using Agencourt AMPure XP beads (Beckman Coulter, USA) following the manufacturer’s instructions. Sequencing was conducted on the Illumina MiSeq platform (Illumina Inc., USA) with a v2 500 kit, which generates paired end reads of 250 bp.

### Taxonomic profiling of bacterial communities

All the data were processed following standard bioinformatics pipelines, including quality control, sequence alignment (with chimera removal), taxonomic classification, operational taxonomic unit (OTU) clustering and community richness analysis (Mothur 1.44.3^9^) using the SILVA database^10^ (release 138.1). Amplicon sequences are available at the European Bioinformatic Institute (EBI) under the EBI Metagenomic Database with accession number ERP121357.

Functional abundances were predicted through PICRUSt 2 software^11^ and annotated by KEGG^12–15^. The predicted functional abundances were scaled based on the abundance of taxa from which they were derived. Thus, high taxonomic abundances usually generate high functional abundances.

### Composition of bacterial communities

Richness and bacterial abundance were examined in each sample for α-diversity and β-diversity with multivariance analysis on a relative abundance matrix. Richness, bacterial abundance and microbiome composition were compared through Jaccard similarity based on observed richness using Tree.shared in Mothur^9^.

The α-diversity was evaluated through the observed richness, Simpson’s index and Shannon’s index based on the number of OTUs. Diversity estimates were compared between fields by applying two-way ANOVA with a posterior Tukey test.

To verify the spatial distribution patterns of bacteria and their response to variables, multidimensional ordering was carried out to identify which explanatory variables could affect the variances between location and abundance. A canonical correspondence analysis (CCA) was performed to determine whether a linear or unimodal ordering method could be applied to the data. The highest gradient obtained was greater than 3.0 SD units, which indicated that a restricted method based on a unimodal model was adequate. For that, we used CCA (double scale) with an explanatory matrix (abiotic factors) with general averages for the following: pH, chlorides and sulfates. The communities were tested and inferred by the possible ecological preferences for the explanatory variables, considering the different collection points. For a more conservative approach, it the analysis was carried out with the phyla and the main bacterial genera identified. Thus, a triple plot CCA was performed to better observe how explanatory variables (vectored lines) influence the distribution of samples and locations, represented in the axes. The type 2 scaling parameter was used to observe the position of the maximum values ​​of the variables with the explanatory ones by the average of the orthogonal distance between samples and vectored lines. The analysis was conducted using the Vegan community ecology package in R version 2.5-7.

### Culture media

Specific culture media were used for SRB and APB growth. For APB growth, Phenol Red Broth medium (Merck, Germany) with 15 g L^−1^ glucose was used. The medium was purged with N_2_ to decrease the oxygen content^16^ (1 h per L) and added to 30 mL sealed bottles. The bottles were sterilized in an autoclave for 15 min at 121 °C. The same process was carried out for the SRB growth medium, which was modified Postgate E^17,18^ (KH_2_PO_4_, 0.5 g·L^−1^; NH_4_Cl, 1.0 g·L^−1^; Na_2_SO_4_, 1.0 g·L^−1^; CaCl_2_·6H_2_O, 1.0 g·L^−1^; MgCl_2_·7H_2_O, 1.8 g·L^−1^; sodium lactate, 7.0 mL·L^−1^; yeast extract, 1.0 g·L^−1^; ascorbic acid, 0.1 g·L^−1^; FeSO_4_·7H_2_O, 0.5 g·L^−1^; agar, 1.0 g·L^−1^). The pH was adjusted to 7.6 with NaOH solution, and before the use, 4.0 mL·L^−1^ of a 0.025% resazurin solution previously filtered in a 0.22 µm membrane filter, was added.

### Enrichment of SRB and APB from produced water samples

APB and SRB consortia were enriched in specific media for 21 days at 35 °C (average oilfield temperature). APB and SRB consortia were transferred weekly (10% (v/v)) to sterile sealed bottles containing Phenol Red Glucose Broth and modified Postgate E, respectively. After inoculation with SRB or APB consortia, a layer of mineral oil was added to maintain the anaerobic environment.

### Minimum inhibitory concentration (MIC) of biocides against SRB and APB

To evaluate the resistance of the consortia against glutaraldehyde, DBNPA, THPS and ADBAC, MIC analyzes were carried out. Concentrations of 25, 50, 100, 250 and 500 mg·L^−1^ of the biocides were dosed in 96-well microtiter plates^19^ containing specific culture medium and an inoculum of 1 × 10^6^ cells/mL, quantified by a luminometer (Photonmaster, Luminultra) using a specific reagent kit (ATP Quench Gone Aqueous Kit, Luminultra). A positive control of each consortium was used for the validation of the test. The bottles were incubated in an anaerobic jar with an anaerobiosis generator (Anaerobac, Probac, Brazil) for 72 h at 35 °C.

## Results and discussion

### Produced water characterization

In the O&G industry, produced water is the term used for water emulsified with oil during the extraction process, and due to the large volumes generated, it is considered one of the most important effluents in O&G. In 2009, the world generation of produced water was estimated to be > 70 billion barrels per year. In Brazil, the average generation is approximately 224 million barrels per day^20^. Worldwide, the volume of produced water is increasing due to the maturation process of reservoirs and the greater number of production fields ^21,22^.

Produced water presents a variable composition according to the geographic location of the field, age and depth of the well, composition of the oil, and extraction method^21^. Despite the proximity of the onshore fields where the samples were collected (Fig. 1), their chemical and biological characteristics were very distinct. Onshore 1, approximately 50 km from Onshore 2, showed higher salinity and sulfate contents but lower pH and microorganism concentrations. The offshore samples presented more similar characteristics (except sulfate content), as expected, since they were collected from the same platform but different wells (Table S1—supplemental material).

### Composition of the bacterial communities from produced water samples

The sequencing produced a total of 438,361 sequences from the produced water samples. After removal of primers, sequences not belonging to the hypervariable V4 region, preclusters and chimeras, 263,241 sequences were used for taxonomic identification. Using Mothur for removal, 258,741 bacterial sequences were filtered, of which 29,929 were unique sequences. A total of 2,636 OTUs were obtained, covering a maximum distance of 3% for the 16S rRNA gene. The coverage was evaluated by a rarefraction curve (Figure S1A – supplemental material), where a trend in the reduction of the curve slope was observed, demonstrating that the majority of OTUs were sampled but sometimes not detected. Indications of alpha diversity showed significant differences (p < 0.0001) between the collection sites. The Chao 1 index showed that most samples presented differences, except Offshore 1a and Onshore 1 (p = 0.8471). The Shannon index did not show significant differences between the samples (p > 0.9999) (Figure S1B—supplemental material). These data demonstrate that bacterial communities do not share rare species in an expressive way. OTUs from Archaea were found in low number (less than 0.1%) in the samples.

**Figure 2:**
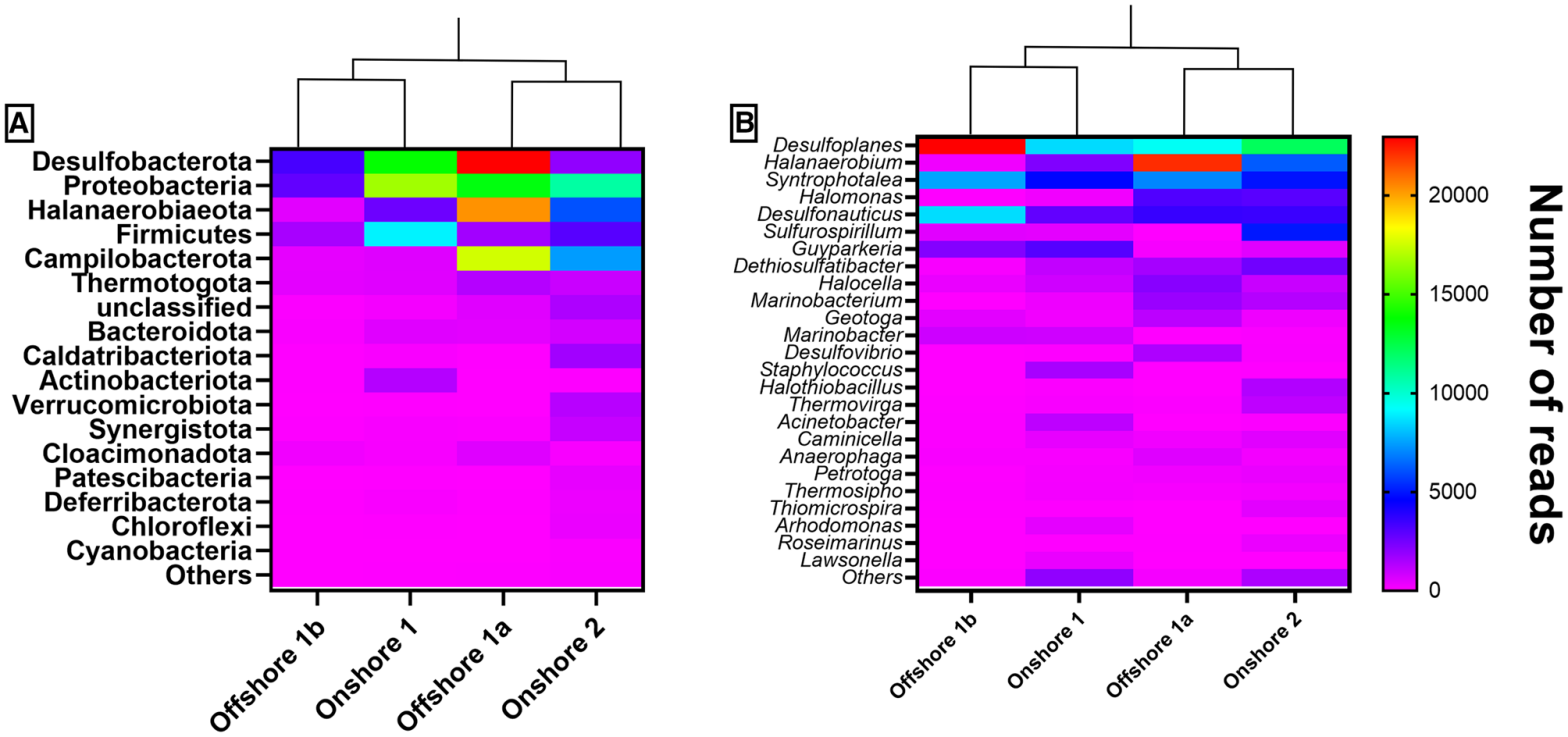
Heatmap of taxonomic analysis at the phylum level (**A**) and genus level (**B**).

Similar to the physical–chemical characteristics, the microbiome of produced water changed according to the sample collection location. However, some microorganisms with important ecological roles have been frequently reported. Sulfate- and sulfur-reducing, acid-producing, thermophilic, halotolerant and halophilic bacteria are the most common groups comprising oil reservoir ecosystems^5,6^. Phyla and genera belonging to these groups were also identified in the produced water samples evaluated in this study. Figure 2A shows the relative abundance of bacterial phyla found in the samples. The results demonstrate the presence of Desulfobacterota, Proteobacteria, Halanaerobiaeota, Firmicutes and Campylobacterota as the most abundant phyla. The relative abundance of genera can be seen in Fig. 2B; *Desulfoplanes* (SRB), *Halanaerobium* (APB), *Syntrophotalea* (sulfur-reducing), *Halomonas* (APB)*, Desulfonauticus* (SRB)*, Sulfurospirillum* (sulfur-reducing) and *Guyparkeria,* also named *Halothiobacillus (*sulfur-oxidizing), were predominant. The identification of these groups of microorganisms can be explained by the extreme conditions found in oilfields, such as high sulfur derivative contents in water, high salinity and high temperatures.

Among the microorganisms found in the samples, SRB are generally the main target in biocidal treatments due to the negative impact caused by H_2_S generation and increased corrosion rates^23–25^. Produced water normally provides favorable conditions for the development of SRB due to the concentrated supply of sulfate and low oxygen content^26,27^. All samples evaluated in this work demonstrated a high abundance of SRB. These microorganisms are able to use sulfate (SO_4_^2−^) as an electron acceptor in dissimilatory sulfate reduction for anaerobic respiration processes, generating H_2_S and other byproducts^27,28^.

In the analyzed samples, the sulfate content was highest in samples Onshore 1 and Offshore 1a (Table S1—supplemental material). Although sulfate plays an important role in the development of the SRB group, the sulfate content was not the only factor affecting the microbiome. Other factors, such as salinity, geographic location, and bacterial competition, influence the predominance of some genera over others.

Similar to SRB, sulfur-reducing bacteria generate H_2_S but use only sulfur (S^0^) and oxidized forms as electron acceptors (except sulfate). Bacteria from sulfur-reducing groups are able to grow through the dissimilatory reduction of elemental sulfur to sulfide in a respiratory type of metabolism ^29,30^ and are frequently found in extreme environments, such as coal mine wastewater and heavy metal-containing waters ^31,32^. Despite their similarities with the SRB group, sulfur-reducing bacteria show important differences in taxonomy^33^, suggesting that organic substrates suitable for SRB may not be suitable for their growth^34^. This group of microorganisms seems to have a key role in the balance of determinate microbiomes, but in most environments, especially in oilfield wastewaters, their role remains unclear.

APBs such as *Halanaerobium* and *Halomonas* intrinsically enter seawater due to their halophilic characteristics and are involved in corrosive processes^35–38^. *Halanaerobium,* predominant in Offshore 1a, is a strictly anaerobic and moderately halophilic genus that requires NaCl concentrations between 30,000 and 200,000 mg L^−1^ for optimal growth^39^. The role of APB microorganisms in biocorrosion has been demonstrated, mostly due to their capacity for biofilm formation ^38,40^ and the production of acids from sugars^6,41^. In addition, studies have demonstrated the synergistic role of APB in SRB biofilms, where the presence of both groups enhances biofilm aggressiveness ^23,42^. Another APB identified in the Offshore 1a sample was *Geotoga*. Despite the low relative abundance in this sample, *Geotoga* has been identified in oil reservoirs^43,44^ and fuel storange tanks^45^.

Another abundant genus found in the analyzed samples was *Syntrophotalea,* also named *Pelobacter*^46,47^*. The Syntrophotalea* genus consists of strictly anaerobic gram-negative bacteria whose members are unable to ferment sugars, metabolizing only an extremely limited number of substrates. *This genus* plays an important role in the fermentative degradation of unusual organic matter, such as ethanol and butanol, and is able to use sulfur (S°) as an electron acceptor in these reactions^32^. In addition, the association of *Syntrophotalea* species with methanogenic bacteria in the degradation of crude oil alkanes, an intrinsic substrate from oil reservoirs, was observed^48,49^.

A significant number of unclassified genera (38% in the four samples), especially those collected from onshore fields, and the high number of OTUs demonstrated the high complexity of the microbiome in produced water. In addition to environmental factors, such as temperature, pressure and geographic location^21^, the microbiome complexity may be influenced by the water composition.

**Figure 3:**
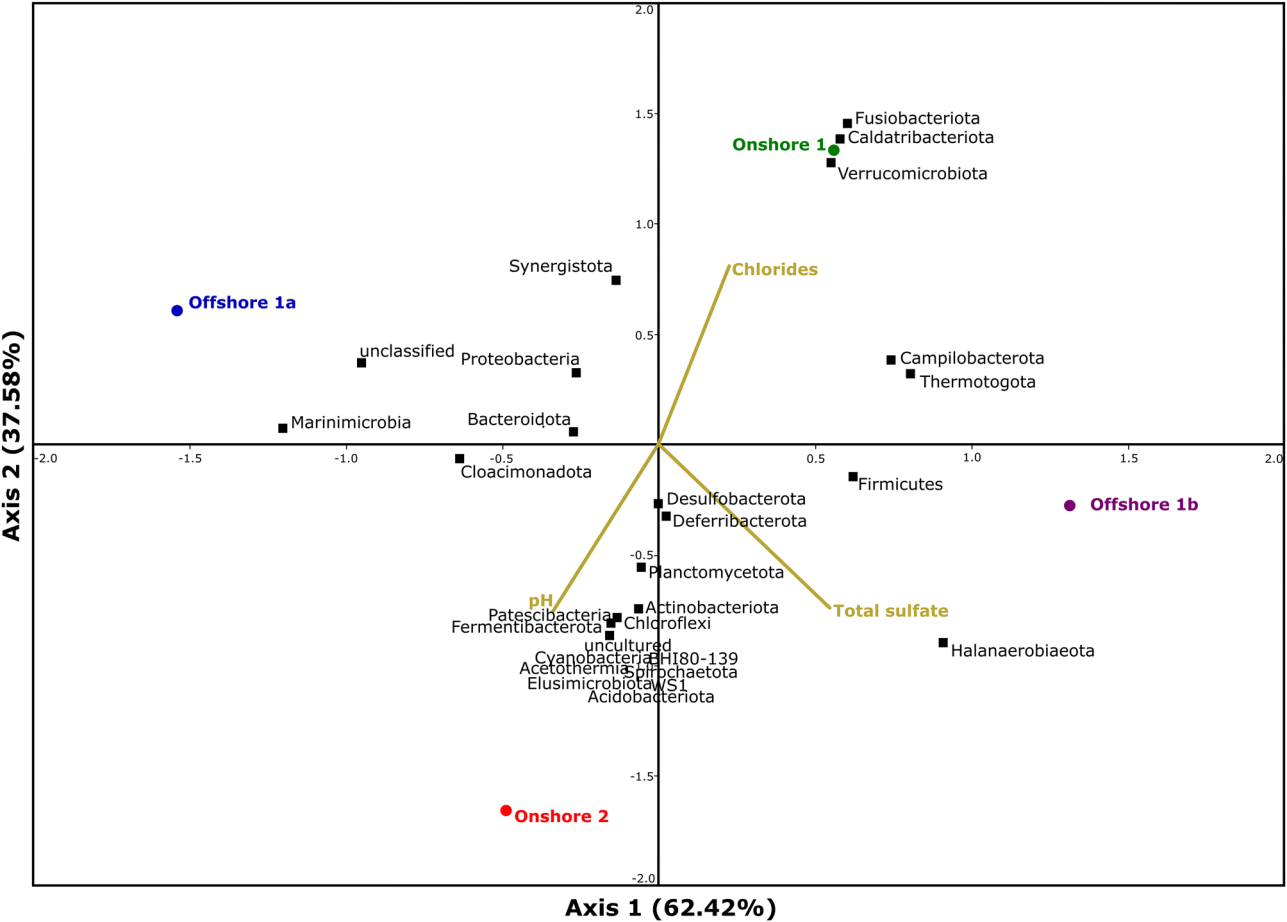
Ordination tri plot of canonical correspondence analysis (CCA) between study sites and abiotic factors and phyla.

**Figure 4:**
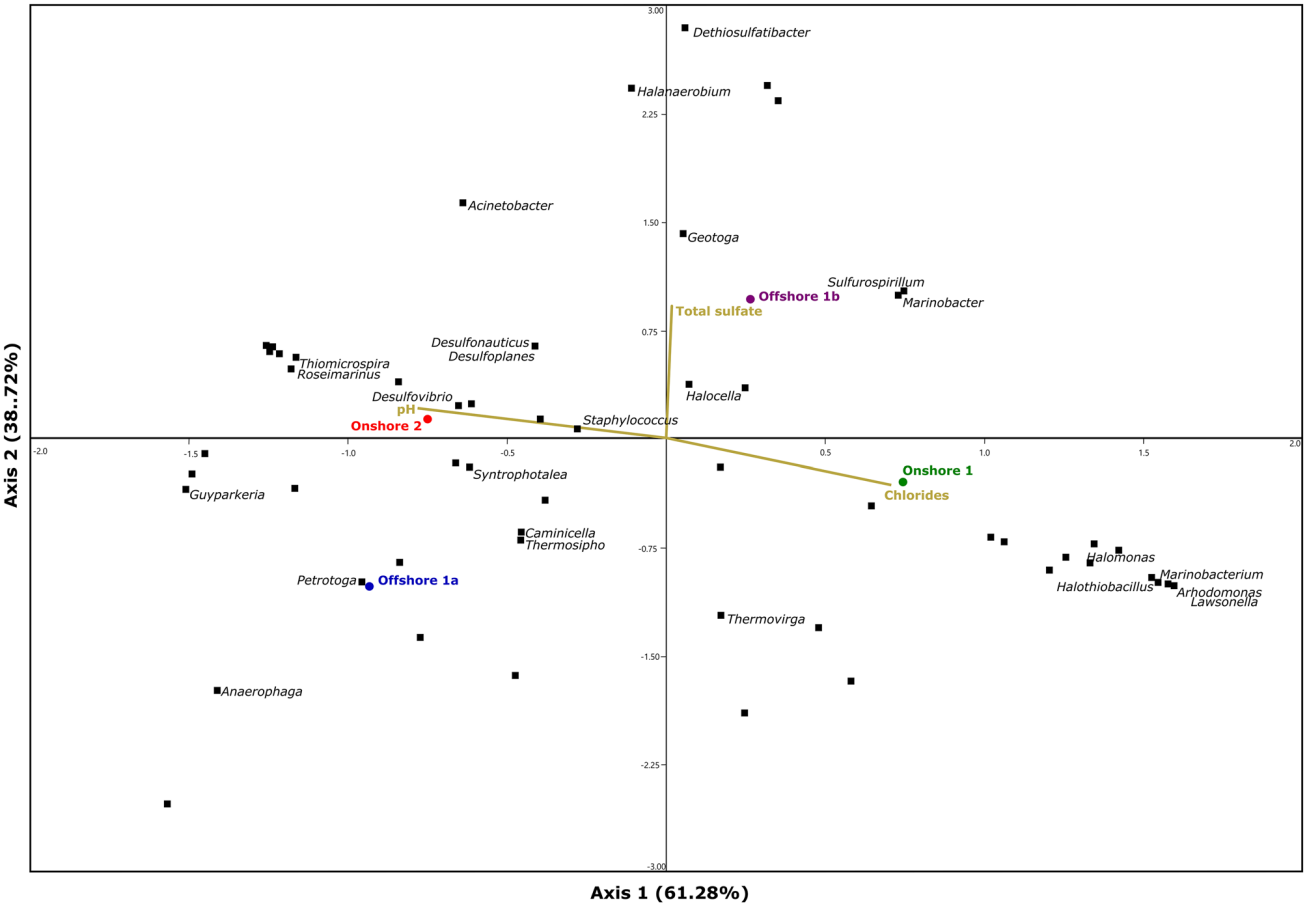
Ordination tri plot of canonical correspondence analysis (CCA) between study sites and abiotic factors and genera.

Figures 3 and 4 show CCA plots of phyla and genera, respectively, according to the abiotic factors associated with the produced water composition that may interfere with microbiome complexity (pH, sulfate and chloride content). Samples Offshore 1b, Onshore 1 and Onshore 2 showed correlations with the factors pH and sulfate content. Desulfobacterota, the most representative SRB phylum found in the four samples, was influenced by these two factors. In addition, although much less abundant, some known APB phyla also demonstrated correlations with pH and sulfate content (Fermentibacterota, Acidobacteriota and Acetothermia). In terms of genera, pH was the most influential, and *Desulfovibrio*, *Desulfoplanes* and *Desulfonauticus* were influenced by this factor.

On the other hand, produced water is a complex matrix, where a small group of abiotic factors cannot reduce all other influences. In this case, factors not evaluated in this work may have contributed to the Onshore 1a microbial composition. Extreme conditions, such as pressure and temperature, and even the selective pressure caused by chemical agents applied in oil fields are examples of possible interfering abiotic factors.

### Functional characterization of the microbiome from produced water

**Figure 5:**
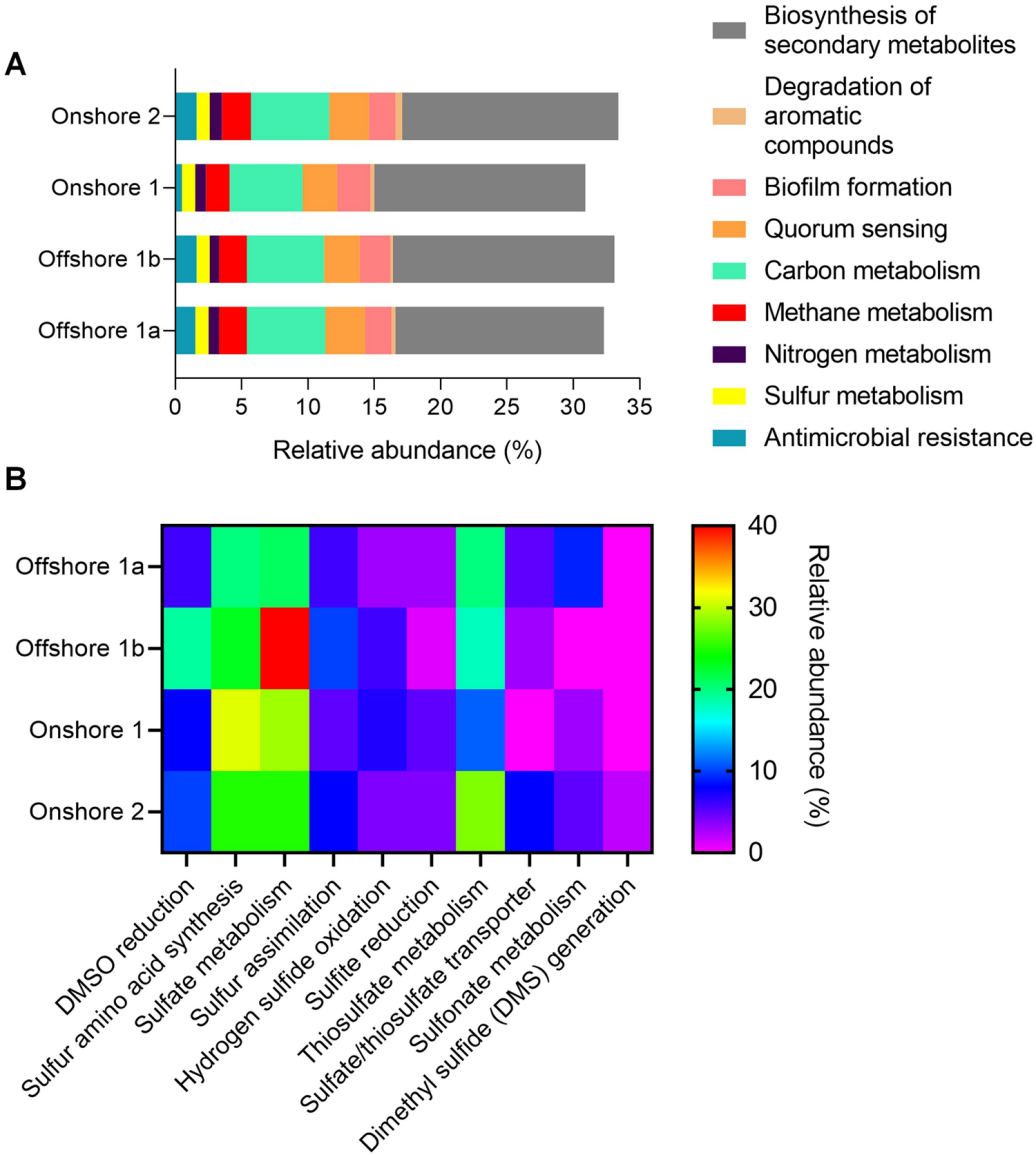
Predictions using KEGG Database of functional abundance orthologous classes (**A**) and genes involved in sulfur metabolism (**B**).

Through the taxonomic abundance of OTUs, the metabolic abundances were predicted. Despite a quite different microbiome taxonomy, the four samples showed remarkably similar metabolic activities (Fig. 5A).

As expected, primary metabolism, for maintenance of bacterial life, was the most abundant in all samples; however, biosynthesis of secondary metabolites also demonstrated substantial relative abundance. Secondary metabolites are produced for survival under certain conditions, such as microbial competition for nutrients or space and protection against abiotic stress^50,51^. Secondary metabolites, such as pantothenate, fatty acids, prodigiosin, biotin, cofactors and vitamins, inosine monophosphate, among others were predicted by KEGG according to microbial composition data.

Other important metabolic activities found were quorum sensing, biofilm formation, carbon, sulfur, nitrogen and methane metabolism, antimicrobial resistance and degradation of aromatic compounds. Quorum sensing and biofilm formation are related to population density control and response to stress and can also be associated with substrate limitation or other types of stress^52,53^. These metabolic activities have been described as a trigger for biofouling in oil fields, causing damage to membrane units, clogging pipes and harming systems and processes^54,55^.

Carbon metabolism is essential for microbial growth due to its activity as an electron donor for respiration, and it is incorporated into cells as biomass. Usually, volatile fatty acids (VFAs) are the most common carbon sources in produced water, but hydrocarbons, including aromatic compounds, have also been found^56^. Initial hydrocarbon degradation presents high energy barriers to be overcome due to the apolar C–H bond. *Pelobacter, Desulfovibrio* and other SRB are part of complex communities involved in anaerobic degradation of hydrocarbons under syntrophic conditions^57^.

Metabolism of sulfur and its derivatives has been frequently associated with negative impacts in oil fields and can increase biosouring in oil reservoirs (biosynthesis of H_2_S)^55,56,58^. The main mechanism for H_2_S generation is the dissimilatory sulfate-reduction pathway, where sulfate is converted to sulfite and then to sulfide by an irreversible reaction. Additional studies suggest two possible alternative biosouring mechanisms: 1) thiosulfate reduction, when the medium presents a low sulfate content, or the presence of sulfate-reducing prokaryotes, and 2) sulfide generation through hydrogen and formate metabolization, mostly by the *Desulfonauticus* genus^59^.

Figure 5B shows the main metabolic pathways involved in sulfur metabolism in the produced water samples evaluated in this work. The highest relative abundances were observed for sulfate metabolism, sulfur amino acid synthesis and thiosulfate metabolism, as expected. However, dimethyl sulfoxide (DMSO) reduction seems to be an important method for anaerobic respiration in extremophiles^60,61^.

Despite the low sulfate concentration of Offshore 1b (Table S1—supplemental material), the sample demonstrated the highest abundance of genes involved in sulfate metabolism. This result was derived from the high abundance of the genus *Desulfoplanes* in the sample (Fig. 3). Possibly, an unknown specific condition was promoting the growth of *Desulfoplanes* in Offshore 1b, for example, high concentrations of alternative substrates. In addition to sulfate, *Desulfoplanes* can metabolize fumarate, formate, lactate, and acetate^62^.

According to OTU abundance, metabolic activities related to antimicrobial resistance were present in all samples, with a relative abundance lower than 2% (regarding the total metabolic activities; data not shown). Analyzing the gene ontologies belonging to the observed OTUs, some similarities and differences can be observed among the samples (Table 1).

**Table 1:** Prediction by KEGG Orthology of the relative abundance of antimicrobial resistance genes in produced water samples.

Metabolic process	Predicted gene	Relative abundance (%)^a^			
		Offshore 1a	Offshore 1b	Onshore 1	Onshore 2
Efflux mechanisms	*AcrA*	5.54	6.52	12.88	6.49
	*AcrB*	4.90	6.06	9.55	5.84
	*TolC*	3.39	0.71	8.03	2.82
	*AdeA*	–	–	0.02	0.01
	*AdeB*	–	–	0.02	0.01
	*AdeC*	–	–	0.08	0.01
	*OprM*	–	–	0.27	0.73
Target modification	*mrcA*	7.20	8.81	12.48	7.67
	*mrdA*	7.07	8.85	11.44	7.58
	*ftsl*	4.10	5.97	8.64	5.86
Modification/degradation of antimicrobial compounds	*ALDH*	4.04	3.39	–	3.96
	*ampC*	–	–	0.11	0.03
	*ampG*	1.71	0.68	4.68	1.49
	*penB*	1.39	0.01	1.02	0.76
	*blaZ*	–	–	0.46	–

^a^Abundance relative to total antimicrobial resistance mechanisms.

Antimicrobial efflux mechanisms were present in the four samples and can include several types of efflux systems. The resistance-nodulation-division system (RND) operates as part of a tripartite system and is found ubiquitously in bacteria, archaea and eukaryotes. RND pump ontologies, such as AcrA/AcrB/TolC, MexAB/OprM and AdeABC systems, were widely found in microorganisms from produced water samples. These systems can extrude multiple antimicrobials using energy conversion from the proton motive force due to their broad substrate specificity^63–65^. Vikram et al., 2015 described efflux systems as the main resistance mechanism used by *Pseudomonas fluorescens* biofilms against glutaraldehyde. A comparison of the response to biocides of glutaraldehyde- and PBS-exposed biofilms at the transcriptomic level revealed the induction of genes related to RND systems and ABC transporters^66^.

Mechanisms related to target modification identified in the microbiome of produced water samples are derived from penicillin-binding proteins (PBPs), which, in addition to their role in cell wall synthesis, precisely in peptidoglycan crosslinking, are membrane-associated macromolecules that play key roles in resistance mechanisms^67^. These enzymes are targets of β-lactam antibiotics, but conformational modifications can lead to the nonrecognition of the active site by antibiotics and, consequently, to antimicrobial resistance. PBP ontologies found in the samples indicated higher abundances of the genes *mrcA*, *mrdA* and *ftsl*. Despite the cross-resistance between antibiotics and biocides being studied^68^, the role of PBP in resistance to biocides, specifically in oil fields, remains unclear.

The modification/degradation of antimicrobials by microorganisms can occur through the action of enzymes such as aldehyde dehydrogenase (ALDH), which catalyzes the oxidation of aldehydes, commonly present in biocides, to carboxylic acids. These enzymes are also involved in several other important metabolic processes, including glycerol production and ethanol oxidation^69^. ALDHs from different microorganisms show different substrate specificities but are able to degrade biocides such as glutaraldehyde and formaldehyde^70,71^. For these reasons, microorganisms able to produce ALDH enzymes should present advantages in terms of intrinsic resistance against aldehyde-based biocides.

Although present in lower abundance in most samples, β-lactamases, uniquely responsible for β-lactam degradation and resistance, were also present. Genes codifying β-lactamase classes A, B, C and D were found, but those codifying classes C and D demonstrated the highest abundances in the microbiomes.

In general, the microbiome from produced water samples demonstrated a huge variety of pathways involved in primary and secondary metabolism. Studies have shown that microorganisms found in oil fields carry out extremely complex “cross feeding” among them named anaerobic syntrophy^57,72,73^. Due to these microbial interactions and diverse microbiomes, oilfield microorganisms have an interdependent lifestyle, which must be deeply studied to fully understand their metabolic activities.

### Biocidal resistance of SRB and APB consortia

SRB and APB consortia from produced water samples were previously enriched in specific media, except consortia from Offshore 1b and Onshore 1, which did not present cultivable SRB or APB or cultivable SRB, respectively. MICs of biocides commonly applied in produced water treatment (50% glutaraldehyde, 50% THPS, 50% DBNPA and 50% ADBAC; active contents were equalized to facilitate the analysis) were applied to the SRB and APB consortia.

Table 2 shows the MIC results, where the different behaviors of the consortia against the biocides can be observed. The SRB consortia demonstrated higher susceptibility to glutaraldehyde and THPS, but at different levels. Offshore 1a presented a lower MIC during glutaraldehyde exposure (< 25 mg·L^−1^), while Onshore 2 presented reduced susceptibility to glutaraldehyde and THPS (MIC = 100 mg·L^−1^, for both molecules). In general, SRB consortia were less susceptible to DBNPA and presented resistance against ADBAC. SRB resistance to ADBAC and other quaternary ammonium compounds has also been reported in several studies ^74–76^ but needs further study.

**Table 2:** MICs of glutaraldehyde, THPS, DBNPA and ADBAC against APB and SRB consortia from produced water samples in Modified Postgate E medium (for SRB) and Phenol Red Glucose Broth (for APB). + , positive growth; –, negative growth.

Biocide	SRB			APB		
	Dosage (mg·L^−1^)	Offshore 1a	Onshore 2	Offshore 1a	Onshore 1	Onshore 2
Positive control	0	+	+	+	+	+
Glutaraldehyde 50%	25	–	+	+	+	+
	50	–	+	+	+	+
	100	–	–	+	+	+
	250	–	–	+	+	+
	500	–	–	+	+	+
THPS 50%	25	+	+	+	+	+
	50	+	+	+	+	+
	100	+	–	+	+	+
	250	–	–	+	+	+
	500	–	–	+	+	+
DBNPA 50%	25	+	+	+	+	+
	50	+	+	+	+	+
	100	+	+	+	+	+
	250	+	–	–	–	–
	500	+	–	–	–	–
ADBAC 50%	25	+	+	+	+	+
	50	+	+	+	+	+
	100	+	+	+	+	–
	250	+	+	+	+	–
	500	+	+	–	+	–

High levels of APB resistance were reported, and unlike for SRB, only DBNPA and ADBAC showed effectiveness at the tested dosages. Glutaraldehyde and THPS required dosages above 500 mg L^−1^ to exert bacteriostatic effects on the APB consortia.

The use of alternative biocides can reduce microbial resistance, changing the biocidal mechanisms to control microorganisms. Green molecules are becoming a new trend due to their lower environmental impact and renewable sources. Natural oils such as lemon^77^, neem^78^, and clove oils^79,80^ and plant extracts such as those of green tea^81^ and garlic^81^, amino acids^82^ and biosurfactants^83^ have all been successfully evaluated against oilfield microorganisms and biofilms. Another way to broaden the effectiveness of water treatment is the use of synergistic molecules, especially those with different modes of action or cell targets.

## Conclusions

This work revealed the high diversity of extremophiles in the produced water microbiome, even in the samples collected from the same platform. Despite the diversity, SRB, APB and sulfur-reducing bacterial groups were found with high abundance in the four samples. Functional characterization of gene ontologies predicted for the microbiomes demonstrated similar activities, mostly due to the similar nature of the samples. In terms of resistance, the SRB and APB consortia enriched from produced water samples demonstrated varied resistance levels to biocides commonly used in water treatment, indicating a specific taxonomic response to each biocide and reinforcing the idea of the necessity of combined treatments (with more than one molecule) or new molecules to broaden treatment effectiveness. To confirm the findings found in this work, further evaluations to identify the bacterial composition of SRB and APB consortia need to be carried out in addition to further resistance studies.

## Supplementary Information

Supplementary Information.
