# Supplementary material for: The impact of bacterial diversity on resistance to biocides in oilfields
Source: Sci Rep. 2021 Nov 29;11:23027. doi: 10.1038/s41598-021-02494-7 (PMC8630110; doi:10.1038/s41598-021-02494-7)
Supplement: Supplementary file 1 — Supplementary Information. [file 41598_2021_2494_MOESM1_ESM.docx]

**Table S1.** Composition of produced water samples according to physical-chemical characteristics and microbial concentration.

| Sample | Location | Collect point | Chlorides  (mg·L^-1^) | Sulfate (mg·L^-1^) | pH | Cells/mL |
| --- | --- | --- | --- | --- | --- | --- |
| Onshore 1 | Bahia state | Water station entrance | 84,000 | 2,342 | 5.8 | 8.5 × 10^4^ |
| Onshore 2 | Bahia state | Water station entrance | 26,550 | 692 | 6.7 | 1.2 × 10^5^ |
| Offshore 1a | Rio de Janeiro state | Production header A | 38,150 | 3,314 | 6.6 | 2.2 × 10^7^ |
| Offshore 1b | Rio de Janeiro state | Production header B | 30,250 | 983 | 6.6 | 4.2 × 10^7^ |


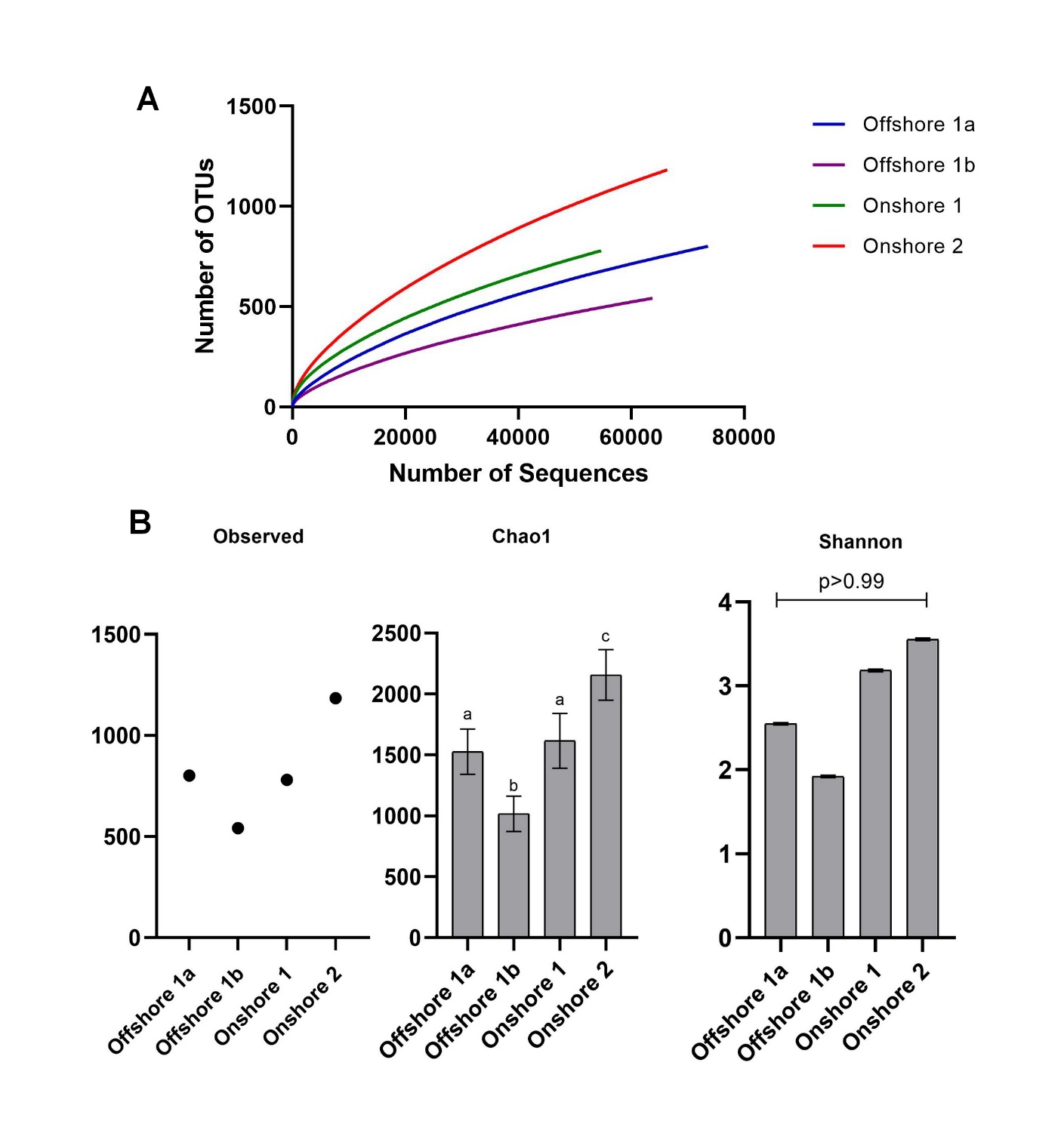


**Figure S1.** Bacterial abundance and diversity in produced water samples. A) Refraction curves of the collection site by the number of sequences. Operational taxonomic units with a maximum distance of 3% for the 16S rRNA gene. B) Results of Alpha diversity analysis (observed richness, Chao1 and Shannon). Different letters represent significant changes between the groups.
